# Autophagy in the pathogenesis and therapeutic potential of post-traumatic osteoarthritis

**DOI:** 10.1093/burnst/tkac060

**Published:** 2023-01-31

**Authors:** Yunquan Gong, Song Li, Jinghui Wu, Tongyi Zhang, Shunzheng Fang, Daibo Feng, Xiaoqing Luo, Jing Yuan, Yaran Wu, Xiaojing Yan, Yan Zhang, Jun Zhu, Jiangyi Wu, Jiqin Lian, Wei Xiang, Zhenhong Ni

**Affiliations:** State Key Laboratory of Trauma, Burns and Combined Injury, Department of Rehabilitation Medicine, Daping Hospital, Army Medical University, Changjiang Street, Yuzhong District, Chongqing 400042, China; Department of Orthopedics, Shanghai Hospital, Shanghai Street, Wanzhou District, Chongqing 404000, China; Department of Wound Repair and Rehabilitation Medicine, Center of Bone Metabolism and Repair, Laboratory for Prevention and Rehabilitation of Training Injuries, State Key Laboratory of Trauma, Burns and Combined Injury, Trauma Center, Research Institute of Surgery, Daping Hospital, Army Medical University, Changjiang Street, Yuzhong District, Chongqing 400042, China; State Key Laboratory of Trauma, Burns and Combined Injury, Department of Rehabilitation Medicine, Daping Hospital, Army Medical University, Changjiang Street, Yuzhong District, Chongqing 400042, China; Joint Surgery and Sport Medicine Department, Hunan Provincial People's Hospital, the First Affiliated Hospital of Hunan Normal University, Jiefangxi Street, Furong District, Changsha 410005, China; State Key Laboratory of Trauma, Burns and Combined Injury, Department of Rehabilitation Medicine, Daping Hospital, Army Medical University, Changjiang Street, Yuzhong District, Chongqing 400042, China; Department of General practice, Chinese PLA General Hospital of the Central Theater Command, Wuluo Street, Wuchang District, Wuhan 430000, China; State Key Laboratory of Trauma, Burns and Combined Injury, Department of Rehabilitation Medicine, Daping Hospital, Army Medical University, Changjiang Street, Yuzhong District, Chongqing 400042, China; State Key Laboratory of Trauma, Burns and Combined Injury, Department of Rehabilitation Medicine, Daping Hospital, Army Medical University, Changjiang Street, Yuzhong District, Chongqing 400042, China; Department of Wound Repair and Rehabilitation Medicine, Center of Bone Metabolism and Repair, Laboratory for Prevention and Rehabilitation of Training Injuries, State Key Laboratory of Trauma, Burns and Combined Injury, Trauma Center, Research Institute of Surgery, Daping Hospital, Army Medical University, Changjiang Street, Yuzhong District, Chongqing 400042, China; Department of Biochemistry and Molecular Biology, College of Basic Medical Sciences, Army Medical University, Gantaoyan Street, Shapinba District, Chongqing 400038, China; Department of Clinical Biochemistry, Faculty of Pharmacy and Laboratory Medicine, Army Medical University, Gantaoyan Street, Shapinba District, Chongqing 400038, China; Department of Clinical Biochemistry, Faculty of Pharmacy and Laboratory Medicine, Army Medical University, Gantaoyan Street, Shapinba District, Chongqing 400038, China; Department of Pediatrics, People's Hospital Affiliated to Chongqing Three Gorges Medical College, Guoben Street, Wanzhou district, Chongqing 404000, China; Department of Cardiology, Shanghai Hospital, Shanghai Street, Wanzhou District, Chongqing 404000, China; Department of Sports Medicine and Rehabilitation, Shenzhen Hospital, Peking University, Lianhua Street, Futian District, Shenzhen 518034, China; Department of Clinical Biochemistry, Faculty of Pharmacy and Laboratory Medicine, Army Medical University, Gantaoyan Street, Shapinba District, Chongqing 400038, China; State Key Laboratory of Trauma, Burns and Combined Injury, Department of Rehabilitation Medicine, Daping Hospital, Army Medical University, Changjiang Street, Yuzhong District, Chongqing 400042, China; State Key Laboratory of Trauma, Burns and Combined Injury, Department of Rehabilitation Medicine, Daping Hospital, Army Medical University, Changjiang Street, Yuzhong District, Chongqing 400042, China

**Keywords:** Autophagy, Post-traumatic osteoarthritis, mTOR, Noncoding RNAs

## Abstract

Autophagy, as a fundamental mechanism for cellular homeostasis, is generally involved in the occurrence and progression of various diseases. Osteoarthritis (OA) is the most common musculoskeletal disease that often leads to pain, disability and economic loss in patients. Post-traumatic OA (PTOA) is a subtype of OA, accounting for >12% of the overall burden of OA. PTOA is often caused by joint injuries including anterior cruciate ligament rupture, meniscus tear and intra-articular fracture. Although a variety of methods have been developed to treat acute joint injury, the current measures have limited success in effectively reducing the incidence and delaying the progression of PTOA. Therefore, the pathogenesis and intervention strategy of PTOA need further study. In the past decade, the roles and mechanisms of autophagy in PTOA have aroused great interest in the field. It was revealed that autophagy could maintain the homeostasis of chondrocytes, reduce joint inflammatory level, prevent chondrocyte death and matrix degradation, which accordingly improved joint symptoms and delayed the progression of PTOA. Moreover, many strategies that target PTOA have been revealed to promote autophagy. In this review,  we summarize the roles and mechanisms of autophagy in PTOA and the current strategies for PTOA treatment that depend on autophagy regulation, which may be beneficial for PTOA patients in the future.

Highlights

- The roles and underlying mechanisms of autophagy in PTOA have been summarized in this article.
- Some strategies including drugs, physical therapy and biological treatments can play their anti-PTOA effects by enhancing autophagy.
- The current research bottlenecks and future directions about autophagy in PTOA have been discussed.

## Background

Osteoarthritis (OA) is a common musculoskeletal disease with pain, disability and economic loss in patients, which is related to a variety of risk factors such as age, joint injuries, obesity, genetic factors and sex [1,2]. Post-traumatic OA (PTOA) is a subtype of OA, accounting for >12% of the overall burden of OA. PTOA is often caused by joint injuries including ligament rupture and intra-articular fracture. It was shown that people who had a knee injury were more prone to develop knee OA compared with those who did not have a knee injury. Moreover, people who suffered ligamentous and meniscal knee injuries had a significantly increased risk of Knee osteoarthritis (KOA) compared with controls. In addition, more than half of patients with fractures of the distal tibial articular surface will develop OA after the trauma [3–5]. Up to now, the pathogenesis of PTOA has still not been fully understood and the current clinical treatments for PTOA have been unsatisfactory [6–9]. Therefore, it is urgent to strengthen the related research to analyze and clarify PTOA pathogenesis so as to provide new strategies for PTOA therapy [10]. The pathological features of PTOA involve the whole joint tissues, including cartilage degradation, subchondral bone remodeling, osteophyte formation and synovial inflammation. Among them, articular cartilage degeneration is the core pathogenesis of PTOA [9,11,12].

Autophagy is a highly conserved metabolic degradation process by which cells recycle substrates to maintain homeostasis [13,14]. Autophagy includes macroautophagy (hereafter referred to as autophagy), microautophagy and molecular chaperone-mediated autophagy as different modes of degradation [15]. After the initiation of autophagy, autophagosomes gradually formed and wrapped the damaged organelles, macromolecular proteins and other substances, then they fused with lysosomes and finally degraded their contents by lysosomal acid hydrolase [16]. The general process of microautophagy is the non-specific or specific encapsulation of substrates by cells through the tonoplast or lysosomal membrane, followed by their degradation in lysosomes [17,18]. Chaperone-mediated autophagy is a process in which HSP70 recognizes specific substrates, then binds to LAMP-2 on the lysosomal membrane and finally transfers the substrates to lysosomes for degradation [19,20]. Autophagy is involved in many physiological and pathological processes including immunomodulation, inflammatory reaction, aging, metabolic diseases and tumors [21–23]. In the past decade, an increasing number of studies have shown that autophagy plays a crucial role in maintaining chondrocyte homeostasis and is a novel potential target for PTOA treatment [24]. This article summarizes the roles and mechanisms of autophagy in PTOA and the current strategies for PTOA treatment that depend of autophagy regulation.

## Review

### Characteristics of PTOA

PTOA is commonly initiated after acute joint damage including anterior cruciate ligament (ACL) rupture, meniscus tear, shoulder dislocation, patellar dislocation, ankle instability or articular surface injuries [10,25,26]. People with experience of joint trauma have an obviously increased risk of PTOA. It was reported that nearly half of the people who suffered from significant joint injury would progress to OA at a later stage [3,25,26]. Because of the different causes of the disease, patients with PTOA are relatively younger than those with aging-related OA. Similar to aging-related OA, patients with PTOA have clinical symptoms of joint pain and motion limitation, which may present as progressive aggravation and even ultimately lead to disability. Although a variety of methods, including surgery, have been developed to treat acute joint injury, the current measures have limited effect on reducing the incidence of PTOA and improving its clinical symptoms in long-term follow-up studies.

In terms of pathological changes, PTOA is characterised by cartilage damage, subchondral bone remodeling, synovitis, meniscus injury etc., which are similar to those in aging-related OA. As to the mechanism, the abnormality of the biomechanics and the inflammatory response are mainly involved in the pathological process of PTOA. The trauma can result in joint instability, which gradually aggravates the degeneration and injury of cartilage as well as subchondral bone remodeling by influencing the shear and contact stresses on the articular surface [27]. Excessive mechanical loading can promote cartilage degeneration and aggravate OA progression via the gremlin-1-NF-κB pathway [28]. Local delivery of an NF-κB inhibitor following joint injury could reduce chondrocyte death and influence pain-related sensitivity in a non-invasive loading model of PTOA [29]. Apart from the mechanical action, multiple biological responses, especially inflammation, greatly contribute to the pathological destruction of the joint at different stages of PTOA [30–32]. Once the joint is injured, an acute inflammatory reaction appears immediately in the joint cavity, which is characterized by an increased number of immune cells, production of pro-inflammatory factors and activation of the pro-inflammatory signaling pathway. After the acute phase, the inflammatory reaction gradually decreases, but low-level inflammation still exists in the joint cavity of most patients and is closely related to the progression of PTOA. The immune cells in synovial tissue can release pro-inflammatory factors such as interleukin-1β (IL-1β) and tumor necrosis factor-α (TNF-α), which inhibit the repair process of the traumatic joint and further aggravate cartilage damage [33]. Marks *et al*. selected patients who had ACL transection (ACLT) injury for >6 months and investigated the change in the inflammatory cytokine profile of synovial fluid. Their data revealed that the levels of IL-1β and TNF-α are higher in patients with ACL ruptures than in controls and that this change is associated with chondral damage [34]. These pro-inflammatory cytokines can activate the classical NF-κB pathway in different types of cells in the joint cavity such as chondrocytes and synovial cells [35–37]. The activated NF-κB resulted in an increase of inflammatory factors (IL-8, CCL5, IL-1β, IL-6, TNF-α), catabolic factors [matrix metalloproteinase 1 (MMP-1), MMP-13, a disintegrin and metalloprotease with thrombospondin motifs 4 (ADAMTS4), ADAMTS5] and angiogenic factors [vascular endothelial growth factor (VEGF), basic fibroblast growth factor (bFGF)], which further promotes an inflammatory response and cartilage destruction [35,38]. Targeting the NF-κB signaling pathway could be considered as a potential strategy for PTOA therapy [37,39,40].

In addition, metabolic disorders can also affect the inflammatory reaction in the articular cavity, such as obesity-associated inflammation, which further influences the progression of OA induced by aging, trauma etc. [41,42]. The up-regulation of pro-inflammatory cytokines (IL-1β, IL-6, TNF-α) as well as altered levels of adipokines (leptin and adiponectin) are characteristics of obesity-associated inflammation, which are also increased in the process of PTOA [43]. In general, different types of fatty acids have different roles in inflammatory regulation. The saturated fatty acids including arachidonic acid and its derivatives (prostaglandins and leukotrienes) present a pro-inflammatory effect, while the unsaturated fatty acids such as docosahexaenoic acid and eicosapentaenoic acid have an anti-inflammatory function [41]. The levels of fatty acids and their derivatives are often changed in serum and synovial fluid of OA patients compared to controls, which may contribute to systemic or local oxidative damage and inflammatory responses, and ultimately influence the pathological injury of the joint during the OA process. Recently, Kimmerling *et al*. revealed that conversion of omega-6 into omega-3 polyunsaturated fatty acids can reduce the severity of PTOA induced by destabilisation of medial meniscus (DMM), indicating that this strategy may be beneficial for PTOA in obese patients following injury [43]. In brief, the clinical symptoms and pathological changes of PTOA patients are similar to those of other types of OA, but the risk factors and pathological mechanisms have some characteristics of their own.

### Animal models of PTOA

A number of different animal species, e.g. mouse, rat, hamster, pig, cat, rabbit and dog, have been used for OA basic research, among which the mouse and rat are the most common [10,44–46]. The existing models for OA research can be divided into four categories, including surgical model, chemical model, genetic model and naturally occurring model (Table 1). These OA models have their own advantages and disadvantages and are selected for experiments according to the different research purposes [10,45,46]. The surgically induced OA model mainly damages the stability of joints by injuring ligaments, meniscus and other structures, thus inducing the occurrence and progress of OA. This model can simulate the pathological process of PTOA in the clinic and is considered as the best model for studying PTOA [44,45]. In addition, the OA phenotype can be rapidly induced in this model compared with the naturally occurring model. Moreover, this OA model is relatively stable with a high repetition rate. Therefore, the surgically induced OA model, especially the DMM-induced model, has been widely used for studying other types of OA in addition to PTOA. To some extent, the surgical model is used as a general model for OA research at present [44,47]. In this review we mainly included the relevant literature using the surgical model, such as DMM, ACLT, etc.

**Table 1:** Different types of OA models

**Model**	**Method**	**Use**	**Advantage**	**Disadvantage**
Surgical model	1. Destabilization of medial meniscus (DMM). 2. Anterior cruciate ligament transection (ACLT). 3. Partial medial meniscectomy (PMM). 4. Intra-articular tibial plateau fracture. 5. ACLT and removal of medial/lateral meniscus or transection of posterior/medial/lateral collateral ligament.	1. Investigating the pathogenesis of osteoarthritic lesions, especially trauma-induced OA. 2. Assessment of therapeutic efficacy of different OA treatments.	1. OA phenotype can be induced rapidly. 2. The model is relatively stable with a high repetition rate. 3. It is similar to the clinical course of PTOA patients.	As it is generated by traumatic intervention, it is not an ideal model for the study on the pathogenesis of degenerative OA.
Chemical model	1. Intra-articular injection of mono-iodoacetate (MIA). 2. Intra-articular injection of papain. 3. Intra-articular injection of collagenase.	1. Investigating the pathogenesis of osteoarthritic lesions. 2. Assessment of therapeutic efficacy of different OA treatments.	1. OA phenotype is induced the most rapidly. 2. It needs less invasive procedures. 3. It is relatively easy to complete the construction of the model.	It is different from the pathological course of any type of human OA in clinic.
Genetic model	Construction of genetically engineered mice by deletion, mutation and overexpression of the related genes to induce OA occurrence.	Investigating the pathogenesis and molecular mechanism of different types of OA.	1. Through intervention of specific genes, it is helpful to evaluate the impact of molecules and their signaling pathways on OA disease *in vivo*. 2. This model can be applied combined with other OA models.	1. It may cause additional cartilage abnormalities or embryonic lethal deletions. 2. The preparation process of engineered mice is relatively complex and requires certain professional technologies. 3. High experiment cost.
Naturally occurring model	Routine feeding without any treatment.	Investigating the pathological process of degenerative OA	1. The course of spontaneous OA in this model is more similar to that of degenerative OA patients in clinic. 2. Special technical operation is not required.	1. It is time-consuming for OA disease development. 2. The incidence, the location of occurrence and the severity of OA in this model are random, which is unsuitable for the assessment of therapeutic efficacy of intervention strategies.

*OA* osteoarthritis, *PTOA* post-traumatic osteoarthritis

**Figure 1:**
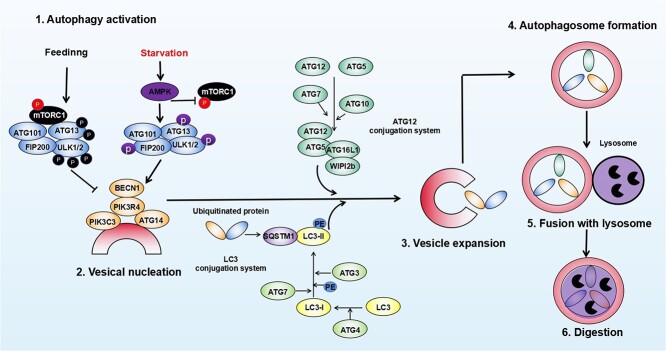
Schematic diagram of the autophagy pathway. Autophagy is precisely orchestrated by different autophagy-related genes (ATGs) and mainly includes the following six key steps. (1) Activation of autophagy. The ULK1/2 complex is activated by inhibiting mTORC1 under starvation. (2) Vesicle nucleation of autophagy. This step begins with the recruitment and activation of class III phosphatidylinositol 3-kinase complex that is composed of BECN1, PIK3R4, PIK3C3 and ATG14. (3) Vesicle expansion of autophagy. In this step, the expansion is assisted by two ubiquitin-like ligation systems. (4) Autophagosome formation. (5) Autophagosome fuses with the lysosome. (6) Digestion of the content. *mTORC1* mammalian target of rapamycin complex 1, *FIP200* FAK family kinase-interacting protein of 200 kDa, *ULK1* Unc-51-like autophagy activating kinase 1, *AMPK* adenosine 5′-monophosphate (AMP)-activated protein kinase, *BECN1* Beclin-1, *PIK3R4* phosphoinositide 3-kinase regulatory subunit 4, *PIK3C3* phosphatidylinositol 3-kinase catalytic subunit type 3, *WIPI2b* WD repeat domain phosphoinositide-interacting protein 2 beta, *LC3* light chain 3, *SQSTM1* sequestosome-1, *PE* phosphatidylethanolamine

### Potential role of autophagy in the pathogenesis of PTOA

Autophagy is precisely orchestrated by different autophagy-related genes (ATGs) and mainly includes six key steps (Figure 1). Firstly, the activation of autophagy depends on inhibition of the activity of mammalian serine/threonine kinase target of rapamycin complex 1 (mTORC1) by AMPK under starvation. Inhibition of mTORC1 results in the dephosphorylation of AGT13 and unc-51-like autophagy activating kinase 1/2 (ULK1/2), and further activating the ULK1/2 complex that consists of AGT13, ULK1/2 and RB1 inducible coiled-coil 1 [48–50]. Subsequently, the activation of the ULK1/2 complex makes the cells progress to the stage of vesicle nucleation of autophagy, beginning with the activation of the class III phosphatidylinositol 3-kinase complex that is composed of Beclin1 (BECN1), phosphoinositide-3-kinase regulatory subunit 4 (PIK3R4), phosphatidylinositol 3-kinase catalytic subunit type 3 (PIK3C3) and ATG14/ATG14L proteins [49,51]. Then, cells come to the stage of vesicle expansion of autophagy, where two ubiquitin-like ligation systems are recruited to conjugate the membrane. One is the ATG5–ATG12 complex formed under the regulation of E1-like enzymes ATG7 and E2-like enzymes ATG10, while the other is the ATG4, ATG7 and ATG3 complex, which transforms light chain 3 (LC3) precursor into an esterified LC3II, leading to the connection of LC3II with the autophagosome membrane. Finally, the autophagosome fuses with the lysosome and the contents are digested and degraded [52,53]. As reported previously, autophagy is often impaired in articular chondrocyte during OA progression. Carames *et al*. demonstrated that the number of autophagic vesicles in articular chondrocytes of 28-month-old mice was significantly decreased compared with 6-month-old mice. At the same time, the expression of autophagy-related genes ATG-5 and LC3 was gradually down-regulated with age, while the level of chondrocyte apoptosis-related markers increased [54]. Carames *et al.* found that the mRNA and protein levels of autophagy marker molecules including ULK1, BECN1 and LC3 were decreased in chondrocytes from OA patients [55]. In addition, Zhang *et al.* found that the expression of autophagy-associated genes is upregulated in animal OA models at the OA early stage compared with that at the late stage, indicating that autophagy is gradually inhibited with the progression of OA [56]. Similarly, the ratio of LC3-II to its free form LC3-I was significantly decreased with the progression of PTOA in an experimental rabbit OA model [57]. Wu *et al*. found that ULK1, one of the autophagy-related genes, was significantly down-regulated in the cartilage of OA patients compared with healthy people [58]. In primary chondrocytes derived from OA patients, ATG5 expression was significantly reduced [59]. In addition, the levels of autophagy-related genes ATG3, ATG4a, ATG5, ATG7 and ATG12 gradually decreased with the progression of OA disease in a rat OA model caused by weight-bearing [56,60,61]. In a rabbit cartilage injury model, the levels of ATG3 and ATG7 were negatively correlated with a cartilage injury [62]. In a monosodium iodoacetate (MIA)-induced cartilage injury model, ATG5 and ATG7 expression were also revealed to be significantly down-regulated [63]. Similarly, in an IL-18 and IL-1-induced chondrocyte injury model, the ATG5 and ATG7 expression level of chondrocytes was also significantly reduced [64,65]. Furthermore, Bouderlique *et al*. employed mice with ATG5-specific knockout in chondrocytes to construct an aging OA model and observed that the ATG5 deficiency led to an increase of cell apoptosis, which in turn aggravated the symptoms of aging OA mice. However, loss of ATG5 in chondrocytes did not regulate PTOA progression in a mouse DMM-induced PTOA model [66]. In addition, a few studies suggested that ATG5 and ATG7 negatively regulated cell death of chondrocyte [67,68]. Furthermore, autophagy can inhibit the expression of chondrocyte catabolism genes and promote the expression of cartilage anabolism genes [69]. Activation of autophagy down-regulated inflammatory catabolic genes such as MMP-3 and -9, ADAMTS5 and CCL-1, -2 and -5 via inhibiting the NF-kB signaling pathway in chondrocytes, which may attenuate the destruction of cartilage [70]. Recently, Zhu *et al*. investigated the effects of meniscal autophagy on the pathogenesis of PTOA. They found that meniscus injury happened prior to the degeneration of articular cartilage in rats after ACLT. In addition,  the secretion from meniscus cells can decrease the levels of MMP13 and ADAMTS5 in IL-1β-treated chondrocytes, indicating that activation of autophagy in meniscal cells may be a potential strategy to delay PTOA progression [71]. Collectively, the key genes regulating autophagy initiation and elongation in chondrocytes are partially inhibited during spontaneous OA or PTOA processes, which negatively influences the homeostasis of chondrocytes and results in cartilage damage via regulation of cell death, cartilage matrix synthesis and inflammatory signaling. However, as the mechanisms of autophagy inhibition and the potential effects of autophagy on cartilage in spontaneous OA and PTOA may be different, further studies on this issue are required in the future.

### Regulation mechanisms of autophagy in PTOA

#### mTOR-dependent regulation of autophagy in PTOA

**Figure 2:**
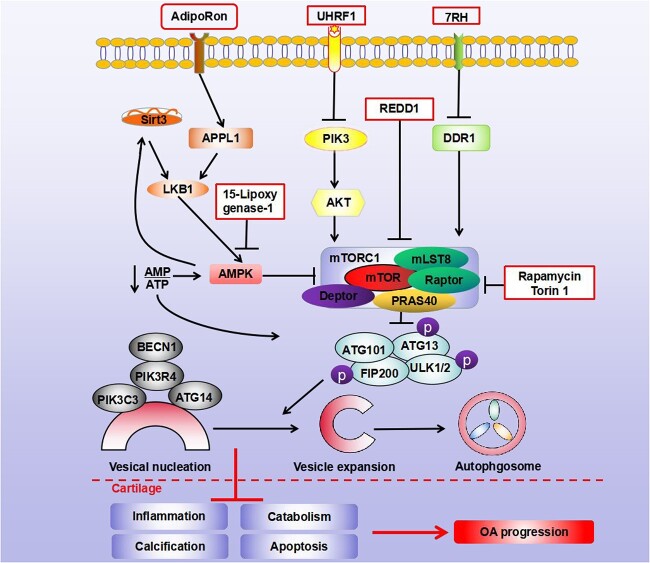
Roles and mechanisms of mTORC1 in PTOA. mTORC1 is inhibited by rapamycin or Torin 1 to activate autophagy, and a protective effect in chondrocytes or cartilage was shown. REDD1, an endogenous inhibitor of mTOR, is thought to be a protector in OA progression by promoting the activation of autophagy. AdipoRon, an adiponectin receptor agonist, targeted the AMPK–mTOR signaling pathway in chondrocytes and was able to activate autophagy and reduce chondrocyte calcification. 7RH, the inhibitor of DDR1 that is located the upstream of mTOR signaling pathway, reduced cartilage degradation and chondrocytes apoptosis by promoting autophagy. 15-Lipoxygenase-1 inhibited AMPK with subsequent activation of mTORC1 and then up-regulated the expression of TGF-beta1 in osteoblasts by regulating the autophagy level. *Sirt3* sirtuin 3, *APPL1* adaptor protein, phosphotyrosine interacting with PH domain and leucine zipper 1, *LKB1* liver kinase B1 homolog, *AMP* adenosine monophosphate, *ATP* adenosine triphosphate, *UHRF1* ubiquitin-like with PHD and ring finger domains 1, *PI3K* phosphatidylinositol 3-kinase, *AKT* serine/threonine kinase 1, *mLST8* mammalian lethal with SEC13 protein 8, *PRAS40* 40 kDa proline-rich AKT substrate, *REDD1* protein regulated in development and DNA damage response 1, *DDR1* epithelial discoidin domain-containing receptor, *PTOA* post-traumatic osteoarthritis

mTOR is a class of macromolecules that mainly participates in the formation of two different complexes, mTORC1 and mTORC2. Of the two, mTORC1 is a well-known target of autophagy inhibition [72–74]. Moreover, upstream signaling pathways such as PI3K/AKT, AMPK and MAPK are regulators of mTOR. The PI3K/AKT pathway in articular cartilage tissue from OA patients was significantly inactivated compared with healthy people [75]. Consistently, the PI3K/AKT pathway was also suppressed in animal PTOA models or injury chondrocytes *in vitro*, which was related to the decrease in ECM synthesis. However, inhibition of mTORC1 by PI3K/AKT can also enhance autophagic flux, which could be beneficial for cartilage homeostasis, suggesting that the PI3K/AKT/mTOR signaling pathway was double-edged for cartilage degeneration and OA progression [76]. Furthermore, Zhang *et al*. employed mice with mTOR specifically knocked out in chondrocytes and uncovered that mTOR deficiency enhanced autophagy activity by mediating ULK1/AMPK activation, thereby improving articular cartilage degeneration, apoptosis and synovial fibrosis in a DMM-induced PTOA model [77]. Carames *et al*. revealed that the mTOR inhibitor rapamycin can induce autophagy, decrease ADAMTS5 and IL-1β levels of chondrocytes and reduced the severity of experimental OA in DMM mice [78]. Besides, Ribeiro *et al*. demonstrated that rapamycin treatment activated autophagy to inhibit chondrocytes’ MMP-13 and IL-12 expression, maintain chondrocyte homeostasis and reduce the damage of cartilage in a DMM-combined diabetic mouse model [79]. Furthermore, activation of autophagy by rapamycin can protect chondrocytes from IL-18-induced apoptosis and improve OA symptoms in a rat PTOA model [64]. Cheng *et al*. used another mTOR inhibitor (Torin 1) to activate autophagy and observed a powerful protective effect of cartilage degeneration in mice after DMM operation [80]. In addition, Alvarez Garcia *et al*. found that the expression level of DNA damage response 1 (REDD1), as an endogenous inhibitor of mTOR, was decreased during OA, while overexpression of REDD1 activated autophagy by inhibiting mTOR signaling [81]. Furthermore, REDD1 deficiency aggravated OA symptoms in articular cartilage, meniscus, subchondral bone and synovium of a mouse PTOA model [82]. Additionally, Xue *et al*. found that enhancing autophagy activity by inhibition of PI3K, AKT and mTOR signaling pathways significantly reduced the inflammatory response of chondrocytes *in vitro* [65]. Duan *et al*. demonstrated that the adiponectin receptor agonist AdipoRon that targets the AMPK-mTOR signaling pathway in chondrocytes can activate autophagy and significantly reduce chondrocyte calcification [83]. Discoidin domain receptor 1 (DDR1) is a kind of transmembrane receptor that can regulate cell proliferation, differentiation and death. In an ACLT surgery-induced mouse PTOA model, DDR1 inhibitor (7rh) reduced cartilage degradation and reduced chondrocyte apoptosis by decreasing mTOR expression and promoting autophagy [84]. 15-Lipoxygenase-1 can upregulate the expression of TGF-beta1 of osteoblasts by regulating the autophagy level in human OA, which was involved in inhibition of AMPK and the subsequent activation of mTORC1 [85]. The above studies suggest that mTOR and its related pathways are closely involved in autophagy activation of OA-related cartilage, which further affects the progression of PTOA and is a promising target for the prevention and treatment of PTOA (Figure 2).

#### Noncoding RNAs related to autophagy in PTOA

Non-coding RNAs are a large class of RNAs without protein-coding ability, including microRNAs (miRNAs), long non-coding RNAs (lncRNAs) and circular RNAs (circRNAs), which function in cell growth, differentiation, senescence and apoptosis. Recent studies have shown that non-coding RNAs actively participate in the onset and progression of OA by regulating chondrocyte autophagy [86–88] (Table 2).

**Table 2:** Non-coding RNAs including miRNAs, lncRNAs and circRNAs that are related to autophagy regulation in PTOA

	**No.**	**Name**	**Model**	**Targets**	**Biological effect**	**Ref.**
miRNA	1	miR-206	1. Primary chondrocytes from Wistar rats 2. Papain and ammoniac-induced OA in rats	IGF-1	Promoting autophagy activity; regulating OA disease process	[90]
	2	miR-4262	TNF-α-treated chondrocytes	SIRT1	Inhibiting chondrocyte autophagy; promoting cell apoptosis	[91]
	3	miR-375	1. Primary chondrocytes from OA patients 2. DMM-induced PTOA in mice	ATG2B	Inhibiting autophagy; promoting endoplasmic reticulum stress	[92]
	4	miRNA-411	IL-1β-treated chondrocytes	HIF-1α	Promoting chondrocyte autophagy activity	[93]
	5	miR-27a	IL-1β-treated chondrocytes	PI3K	Promoting autophagy; inhibiting apoptosis	[94]
	6	miR-107	ACLT with EMM-induced PTOA in rats	TRAF3	Promoting autophagy; reducing cartilage damage	[95]
	7	miRNA-335-5p	Primary chondrocytes from OA patients	—	Promoting autophagy; relieving chondrocyte inflammation	[96]
lncRNA	1	LncRNA-CIR	1. IL-1β-treated chondrocytes 2. MCLT- and MMT-induced PTOA in rats	—	Promoting autophagy; alleviating OA symptoms	[99]
	2	HOTAIR	Cartilage from OA patients	miR-130a-3p	Inhibiting autophagy; promoting cell apoptosis	[100]
	3	SNHG7	IL-1β-induced chondrocytes	miR-34a-5p	Inhibiting autophagy; promoting cell proliferation	[101]
	4	GAS5	Primary chondrocytes from OA rats 2. ACLT-induced PTOA in rats	miR-144	Inhibiting autophagy; promoting cell apoptosis	[102]
circRNA	1	Hsa_circ_0037658	1. IL-1β-treated chondrocytes 2. DMM-induced PTOA in rats	—	Promoting autophagy; inhibiting cell apoptosis	[106]
	2	CircPan3	ACLT- and PMM-induced PTOA in rats	miR-667-5p	Promoting autophagy; reducing matrix metalloproteinases level	[107]
	3	CiRS-7	DMM-induced PTOA rats	miR-7	Inhibiting autophagy; aggravating cartilage degradation	[108]

*miRNA* microRNA, *lncRNAs* long noncoding RNAs, *circRNAs* circle RNAs, *TNF-α* tumor necrosis factor alpha, *DMM* destabilization of medial meniscus, *PTOA* post-traumatic osteoarthritis, *IL-1β* interleukin 1 beta, *MCLT* medial collateral ligament transection, *MMT* medial meniscal tear, *ACLT* anterior cruciate ligament transection, *PMM* partial medial meniscectomy, *EMM* excision of medial meniscus, *IGF-1* insulin-like growth factor 1, *SIRT1* sirtuin 1, *ATG2B* autophagy related protein 2 beta, *HIF-1α* hypoxia inducible factor-1 alpha, *PI3K* phosphatidylinositide 3-kinases, *TRAF3* TNF receptor-associated factor 3

#### miRNAs

miRNAs are a class of endogenous small RNAs of ~20–24 nucleotides in length [89]. In animal OA models, Yu *et al*. described that the miR-206 level was upregulated in a rat OA model. Moreover, miR-206 overexpression reduced autophagy activity and delayed the cell cycle by targeting Insulin-like growth factor-1 (IGF-1), which further activated the PI3K/AKT/mTOR signaling pathway [90]. Sun *et al*. showed that miR-4262 expression was increased in TNF-α-induced chondrocytes. Overexpression of miR-4262 activated the PI3K/AKT/mTOR signaling pathway via targeting SIRT1, which further repressed chondrocyte autophagy and promoted cell apoptosis [91]. Li *et al*. discovered that miR-375 was highly expressed in cartilage of OA patients. miR-375 mimics transfection can inhibit autophagy by down-regulating the expression of ATG2B in chondrocytes, further promoting ER stress and leading to cartilage damage in DMM-induced PTOA mice [92]. Yang *et al*. showed that miRNA-411 could enhance chondrocyte autophagy activity by regulating the expression of HIF-1α in an IL-β-induced chondrocyte injury model [93]. Chen *et al*. also found that miR-27a was involved in the pathogenesis of OA by regulating the PI3K/Akt/mTOR signaling pathway [94]. In addition, Zhao *et al*. found that the content of miR-107 was reduced in OA chondrocytes, and overexpression of miR-107 can inhibit the AKT/mTOR and NF-κB signaling pathways by targeting TNF receptor associated factor 3 (TRAF3), thereby activating autophagy and reducing cartilage damage in a PTOA rat model [95]. Moreover, Zhong *et al*. also suggested that miRNA-335-5p significantly alleviated the inflammation of human OA chondrocytes and inhibited chondrocyte apoptosis by activating autophagy [96]. In brief, the change of miRNAs can affect the degeneration of chondrocytes by inhibiting the expression of autophagy-related genes.

#### lncRNAs

lncRNAs are non-coding RNAs with a length of >200 nucleotides that are involved in many processes of physiology and pathophysiology [97,98]. Increasing evidence has shown that lncRNAs also played an important role in OA. A study by Wang *et al*. demonstrated that lncRNA-Cartilage injury related (CIR) was abundantly expressed in the joint tissue of OA patients, while ATGs including LC3BI/II and BECN1 were up-regulated. Moreover, treatment with si-lncRNA-CIR (Small interfering long no-coding) in mouse joints significantly activated autophagy and alleviated OA symptoms in a PTOA animal model [99]. Furthermore, He et al. discovered that the content of lncRNA HOTAIR was increased in KOA patients’ tibial plateau cartilage tissue. Mechanically, HOTAIR adsorbed miR-130a-3p to inhibit autophagy, leading to cell apoptosis. Conversely, the silencing of HOTAIR reversed the above effects [100]. Tian *et al*. observed that the expression of lncRNA SNHG7 was down-regulated in the serum and joint tissues of OA patients. In addition, it was suggested that SNHG7 participated in the regulation of chondrocyte autophagy and apoptosis by adsorbing miR-34a-5p [101]. In a ACLT-induced PTOA model, the expression of lncRNA GAS5 was notably increased in cartilage tissue. Moreover, overexpression of GAS5 in chondrocytes significantly activated the mTOR signaling pathway, which in turn inhibited autophagy and promoted cell apoptosis [102]. The above research shows that lncRNAs, which were traditionally considered as ‘junk RNA’, can also participate in the maintenance of chondrocyte homeostasis by regulating its target miRNA. Up to now, the research on lncRNAs in PTOA has been relatively limited, and the specific involvement of lncRNAs in cartilage degeneration through regulating autophagy of different cells remains to be futher studied.

#### circRNAs

circRNAs are a class of non-coding RNA molecules without a 5′ end cap and a 3′ end poly(A) tail and form a circular structure with covalent bonds. circRNAs are broadly present in organisms and are involved in the transcriptional and post-transcriptional regulation of gene expression [103,104]. Recently, researchers have shown that circRNAs are also involved in OA progression [105]. Sui *et al*. found that hsa_circ_0037658 was significantly up-regulated in cartilage tissue from OA patients. Besides, autophagy could be activated by silencing hsa_circ_0037658 in IL-1β-treated CHON-001 cells [106]. Zeng *et al*. discovered that circPan3 was notably reduced in IL-1β-induced chondrocyte. Furthermore, overexpression of circPan3 in chondrocytes activated autophagy by targeting miR-667-5p, which reduced the MMP level of chondrocytes [107]. Zhou *et al*. discovered that ciRS-7 expression was down-regulated in IL-1β-induced chondrocytes, resulting in an increase in miR-7, which activated the PI3K/AKT/mTOR signaling pathway and inhibited autophagy, ultimately aggravating IL-1β-induced cartilage degradation. Moreover, knockout of miR-7 significantly attenuated DMM-induced cartilage destruction, while miR-7 mimics accelerated OA progression in a mouse PTOA model [108]. In recent years, an increasing amount of evidence shows that circRNAs play an important role in the aging process. circRNAs are relatively stable *in vivo* and have been considered as a potential target for the treatment of degenerative diseases. More studies about the effects and mechanisms of circRNAs during PTOA are needed in the future.

#### Transcription factors that regulate autophagy in PTOA

Transcription factors play a crucial role in the regulation of gene expression [109]. Studies have shown that multiple vital transcription factors are involved in the onset and progression of OA by regulating the autophagy activity of chondrocytes [110]. Wang *et al*. found that ATF4, a fundamental regulator of chondrocyte proliferation and bone formation, protected cartilage and delayed PTOA progression by inducing autophagy with intra-articular injections of ATF4-overexpressing exosomes in a PTOA mouse model [111]. Zheng *et al*. discovered that transcription factor EB (TFEB), a regulator of autophagic flux, was down-regulated in the cartilage of OA patients or animal OA models. Overexpression of TFEB can rescue autophagy level so as to protect chondrocytes against tert-butyl hydroperoxide (TBHP)-induced cell apoptosis and senescence. In addition, TFEB overexpression also enhanced the autophagic flux and ameliorated cartilage degradation in a DMM-induced mouse PTOA model [112]. The Forkhead box protein O (FoxO) family is a class of transcription factors that participate in many aspects of physiology, including stress response, cell senescence, metabolism and cell death. FoxO expression in cartilage decreases with age and OA progression. Matsuzaki *et al*. performed DMM in mice with FoxO1/3/4-specific knockout of chondrocytes to induce PTOA and observed that FoxO transcription factors play a vital role in cartilage homeostasis by promoting autophagy [113]. Moreover, Friedman *et al*. found that A2AR (Adenosine A2A receptor) stimulation increased nuclear localization of FoxO1 and FoxO3, enhanced autophagic flux and reduced apoptosis in chondrocytes [114]. In addition, peroxisome proliferator-activated receptor α (PPARα), as a transcription factor, protected articular chondrocytes from cartilage degradation in LPS-induced chondrocytes as well as in DMM-induced PTOA mice. Zhou *et al*. found that the PPAR α agonist (Wy14643) activated autophagy and increased chondrocyte proteoglycan synthesis, which finally produced chondroprotective effects *in vitro* and *in vivo* [115]. As a major participant in regulating gene expression in cells, the changes of transcription factors may be closely related to PTOA diseases (Table 3). The expression of key autophagy genes driven by transcription factors is very important for the maintenance of chondrocyte homeostasis and microenvironment balance, which could be a potential target for PTOA therapy.

**Table 3:** Transcription factors (TF) that regulate autophagy in PTOA

**TF**	**Experimental model**	**Biological effect**	**Ref.**
ATF4	Anterior part of medial meniscus excision: induced PTOA in mice by the excision of anterior part of medial meniscus. Tunicamycin-treated chondrocytes.	ATF4-overexpressing OA-Exo alleviated articular cartilage damage in surgically induced PTOA mice. ATF4-overexpressing OA; Exo injection partially restored the impaired autophagy in PTOA mice. ATF4-expressing OA-Exo increased ATF4 level, upregulated autophagy and inhibited apoptosis in chondrocyte	[111]
TFEB	PTOA model induced by DMM. TBHP-treated chondrocytes.	TFEB overexpression ameliorated cartilage injury and synovial inflammation in mouse DMM model. TFEB overexpression rescued TBHP-inhibited autophagic flux induced in primary chondrocytes.	[112]
PPARα	PTOA model induced by DMM. LPS-treated chondrocytes.	Intra-articular injection of WY14643 attenuated articular cartilage degeneration, promoted proteoglycan synthesis and enhanced autophagy *in vivo*. Activation of PPARα by WY14643 increased proteoglycan synthesis via up-regulation of autophagy in LPS-treated chondrocytes.	[115]
FoxO (FoxO1/3/4)	DMM-induced PTOA model. Treadmill running-induced OA model. IL-1β-treated chondrocytes.	AcanCreERT-TKO mice developed mild cartilage lesions (2 months) and progressed to full-thickness cartilage defects (5 months) after tamoxifen administration. The severity of OA induced by DMM and treadmill running was obviously higher in AcanCreERT-TKO mice. Overexpression of FoxO1 up-regulated autophagic genes such as LC3b, Sesn3 and Prkaa2, and partially reversed the inflammatory and cartilage catabolic genes in IL-1β-treated chondrocytes.	[113]

*ATF4* activating transcription factor 4, *PTOA* post-traumatic Osteoarthritis, *OA-Exo* exosomes from osteoarthritis mice, *TFEB* transcription factor EB, *DMM* destabilization of the medial meniscus, *TBHP tert*-butyl hydroperoxide, *PPARα* peroxisome proliferators-activated receptors alpha, *LPS* lipopolysaccharide, *FoxO* forkhead box O, *IL-1β* interleukin 1 beta

#### Acetylation is related to autophagy in PTOA

**Figure 3:**
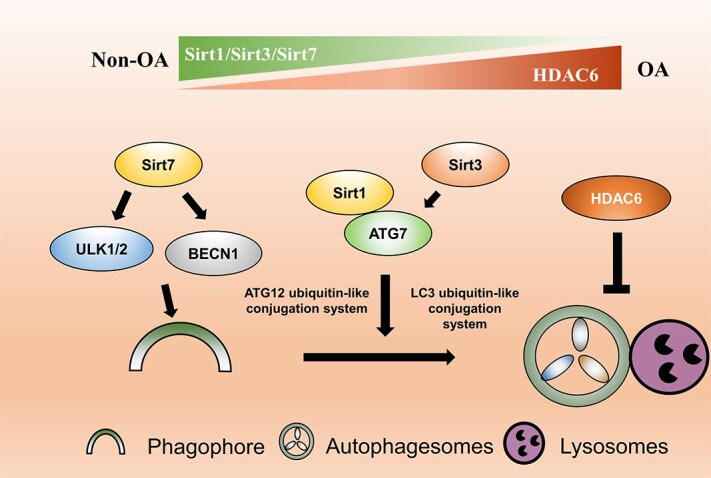
Potential mechanisms of acetylation in PTOA-related autophagy. Sirt7, Sirt1 and Sirt3 were uncovered to enhance autophagy by promoting vesical nucleation and vesicle expansion through regulation of ULK1/2, BECN1 and ATG7 respectively. Inhibition of HDAC6 was demonstrated to promote autophagy activation by accelerating the fusion of autophagosome and lysosome. *ULK1/2* unc-51-like autophagy activating kinase 1/2, *BECN1* Beclin 1, *ATG* autophagy-related gene, *SIRT* sirtuin, *HDAC6* Histone deacetylase 6, *PTOA* post-traumatic osteoarthritis

Acetylation, a process that transfers the acetyl group of acetyl-CoA to protein amino acid residues by acetylase [116], regulates various signaling pathways in cells, including autophagy [117]. Sirtuin (SIRT) is a class of highly conserved deacetylases from bacteria to humans. The human SIRT family consists of seven members, SIRT1–SIRT7 [118]. Wu *et al*. found that SIRT7 was significantly down-regulated in the cartilage of OA patients compared with healthy controls. Further, the level of SIRT7 was also reduced in IL-1β-induced chondrocytes, while overexpression of SIRT7 activated autophagy and protected against cartilage damage [58]. In addition, a study by Liao *et al*. showed that SIRT1 expression was inhibited in cartilage tissue of elderly and OA patients, while up-regulation of SIRT1 can enhance autophagy levels in aged chondrocytes. Mechanically, SIRT1 may regulate chondrocytes autophagy through interaction with autophagy-related ATG7 [119]. Moreover, Sun *et al*. also found that overexpression of SIRT1 in chondrocytes can significantly reduce TNFα-induced cartilage damage [91]. Mitochondrial deacetylase SIRT-3 mediates age-related changes in cartilage via Superoxide dismutase 2 (SOD2)-dependent redox regulation and improves cartilage resistance to reactive oxygen species (ROS), which protects against early-stage OA [120]. Moreover, Xu *et al*. demonstrated that SIRT3 reduced OA joint damage by restoring autophagy influx in a PI3K/Akt/mTOR-dependent manner in a PTOA rat model induced by medial meniscus resection [121]. In addition, HDAC6, as a deacetylase targeting histones, was revealed to be upregulated in a mouse PTOA model, and inhibition of HDAC6 significantly activated cartilage autophagy and reduced cartilage damage [122]. Moreover, Sacitharan *et al*. found that spermidine maintained the homeostasis of chondrocytes through up-regulating acetyltransferase EP300 [123]. Briefly, acetylation can affect the degeneration and destruction of cartilage by regulating the function of autophagy-related proteins (Figure 3). Enzymes that can regulate the acetylation of autophagic proteins, including acetylases and deacetylases, could be the candidate targets of anti-PTOA strategies in the future.

#### Other mechanisms involving autophagy in PTOA

Lin *et al*. found that the expression of FBXO21, an E3 ubiquitin ligase, was up-regulated in the chondrocytes of aging and OA patients. Knockdown of FBXO21 promoted autophagy and reduced chondrocyte apoptosis [124]. Shi *et al*. reported that ubiquitin-like with Plant homeodomain (PHD) and RING finger domain 1 (Uhrf1), a crucial mediator in genome methylation, was down-regulated in cartilage tissue of OA patients compared to healthy individuals. Moreover, knockdown of Uhrf1 enhanced autophagy capacity and reduced apoptosis by inhibiting the PI3K/Akt/mTOR signaling pathway *in vitro* [125]. Chen *et al*. revealed that autophagy was impaired in OA fibroblast-like synoviocytes (OA-FLS), which was negatively regulated by Methyltransferase 3 (METTL3)-mediated excessive m6A modification. METTL3 can reduce the stability of ATG7 mRNA and inhibit the expression of ATG7 by m6A modification, while silencing of METTL3 can enhance autophagic flux and inhibit the senescence-associated secretory phenotype in OA-FLS. Moreover, intra-articular injection of synovium-targeted METTL3 Small interfering RNA (siRNA) activated autophagy of OA-FLS, leading to a decrease in cell senescence and cartilage destruction in DMM-induced PTOA mice [126]. Wei *et al*. showed that upregulation of transient receptor potential cation channel subfamily V member 5 (TRPV5) stimulated Ca^2+^ influx and inhibited autophagy in the injured chondrocytes of MIA-induced OA rat by detection of calmodulin-dependent protein kinase II (CAMK II) phosphorylation, BECN1 phosphorylation, B-cell lymphoma-2 (BCL2) phosphorylation and the LC3-II/LC3-I ratio. Moreover, TRPV5 inhibitors delayed the progression of joint destruction [127]. Furthermore, in an MIA-induced rat OA model and chondrocyte injury model, Zhu *et al*. reported that agonists of alpha7 nicotinic acetylcholine receptors (alpha7-nAChRs) inhibited NF-κB/NLRP3 inflammasome activation by the ROS/TXNIP pathway [128]. Knockout of alpha7-nAChRs in primary chondrocytes decreased LC3 levels under normal conditions and made the cells more sensitive to MIA-induced apoptosis, which could be related to MIA-induced pain behavior and cartilage degradation [129]. The mRNA level of Lysyl oxidase like 3 (LOXL3) increased in OA patients and OA rats, which was positively correlated with the leptin concentration in joint synovial fluid. Leptin significantly promoted cell proliferation and autophagy in primary chondrocytes. In addition, overexpression of LOXL3 inhibited autophagy of chondrocytes mainly through activating mTORC1 [130,131]. Wang *et al*. showed that upregulation of p63, as a member of the p53 family, inhibits chondrocyte autophagy in OA chondrocytes, leading to OA progression [132]. In conclusion, as an important mechanism for maintaining cell homeostasis, the regulation of autophagy level is involved with a variety of signaling pathways from gene expression to protein modification. An in-depth study of the molecular mechanisms of autophagic changes in chondrocytes will provide strong support for autophagy-based treatment in clinical practice.

### Autophagy-related PTOA therapeutic strategies

**Figure 4:**
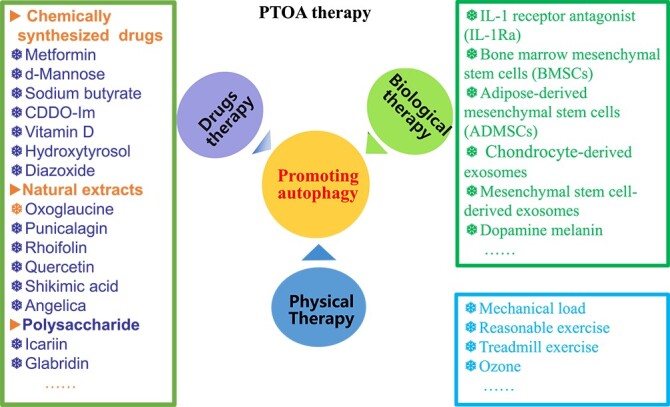
Promoting autophagy by different PTOA therapeutic strategies. Three kinds of strategies for PTOA therapy were related to the enhancement of autophagy, including drug, biological and physical therapy. *IL* interleukin, *PTOA* post-traumatic osteoarthritis

An increasing number of studies have demonstrated that promoting autophagy is an important mechanism for different PTOA treatment strategies, including drugs, physical therapy and biological therapy **(**Figure 4**)**. These strategies can delay the progression of OA by enhancing the autophagy level of chondrocytes, preventing chondrocyte death, attenuating matrix degradation and reducing the inflammatory response.

### Chemically synthesized drugs that target autophagy in PTOA

Metformin, the first-line agent in type 2 diabetes treatment, was reported to increase LC3II, p62 and Lysosomal associated membrane protein 1 (LAMP1) expression levels, which further enhanced the level of autophagy. Na *et al*. reported that oral metformin can protect cartilage tissue and delay OA progression in an MIA-induced rat model [133]. d-Mannose is a monosaccharide with an immunity regulation function and anti-osteoporosis effect through the AMPK pathway that can enhance autophagy activity. Lin *et al*. discovered that d-mannose promoted chondrocyte proliferation and reduced inflammatory factor-induced chondrocyte degeneration *in vitro*. In addition, treatment with a median dose of d-mannose can delay OA progression in MIA-induced mice [134]. Sodium butyrate, a component of short-chain fatty acids, harbors anti-inflammatory and anti-tumor activities by restoring autophagy capacity and increasing autophagic flux by inhibiting the PI3K/Akt–mTOR signaling pathway. Using an IL-1β-induced chondrocyte model and an ACLT mouse model, Zhou *et al*. reported that sodium butyrate (NaB) reduced PTOA chondrocyte inflammation, extracellular matrix degradation and apoptosis and delayed PTOA progression [135]. CDDO-Im is a novel synthetic triterpene and autophagy booster. In a DMM mouse model, Dong *et al*. found that CDDO-Im decreased the release of inflammatory mediators and alleviated knee cartilage erosion. Consistently, CDDO-Im dose-dependently enhanced autophagy *in vitro*, highlighting its cartilage protection and anti-OA activity [136]. Vitamin D, as an anti-osteoporosis drug, plays a vital role in bone health. Kong et al. reported that vitamin D could activate autophagy through the AMPK-mTOR signaling pathway and then inhibit the inflammatory response. In addition, OA symptoms were relieved after vitamin D treatment in a mouse model of PTOA [137]. Hydroxytyrosol has an anti-inflammation function and has been used for treatment of inflammatory diseases. Zhi *et al*. found that hydroxytyrosol can inhibit the inflammatory response of chondrocytes through SIRT6-mediated autophagy [138]. Diazoxide, as one of the potential drugs to prevent OA, can significantly improve the severity of experimental PTOA, which was related to the restoration of impaired autophagy [139]. An increasing number of studies has found that different compounds can cause changes in autophagy levels. However, the mechanisms of these compounds on cells may be complex and autophagy regulation may be one of them. We need to study more compounds with the capacity of relatively specific autophagy activation so as to accurately regulate the autophagic flux in OA and reduce the potential side effects of the compounds.

### Natural extracts that target autophagy in PTOA

The therapeutic effects and detailed mechanisms of natural products have been widely studied in the past decades. Previous studies have suggested that different types of natural products can reduce joint inflammation, prevent cartilage degradation and delay PTOA progression *in vitro* and *in vivo* (Table 4).

**Table 4:** Natural extracts that target autophagy in PTOA

**No.**	**Compound**	**Classification**	**Structure**	**Animal model**	**Biological effect**	**Mechanism**	**Ref.**
1	Oxoglaucine	Alkaloids	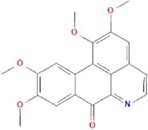	DMM-induced PTOA rats	Accelerating autophagosome formation and preventing cartilage damage by delaying matrix degradation	Blocking Ca^2+^ influx and TRPV5/calmodulin/CAMK-II pathway	[149]
2	Punicalagin	Phenols	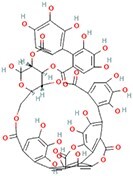	DMM-induced PTOA mice	Activating autophagy and ameliorating OA by inhibiting extracellular matrix degradation	——	[140]
3	Gallocatechin 3-gallate	Phenols	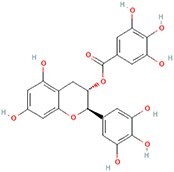	ACLT-induced PTOA rats	Activating autophagy and ameliorating OA by inhibiting chondrocyte apoptosis	Reducing the expression level of mTOR and enhancing the expression of microtubule-associated protein light chain 3, Beclin-1 and p62	[141]
4	Mangiferin	Phenols	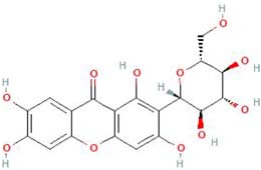	DMM-induced PTOA mice	Restoring autophagy and ameliorating OA by reducing chondrocyte apoptosis and cell-matrix degradation	Activating the AMPK signaling pathway	[142]
5	Icariin	Flavonoids or phenols	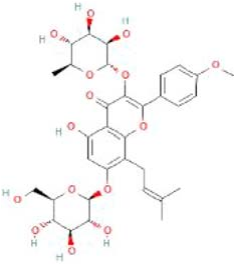	ACLT + MCLT + MX-induced PTOA rats	Activating autophagy and ameliorating OA by decreasing chondrocyte apoptosis	Inhibiting the PI3K/AKT/mTOR signaling pathway	[145]
6	Glabridin	Flavonoids or phenols	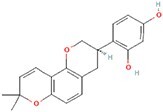	ACLT-induced PTOA rats	Promoting autophagy and ameliorating OA by protecting chondrocytes against oxidative stress and apoptosis	——	[146]
7	Alantolactone	Terpenoids	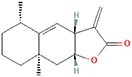	DMM-induced PTOA mice	Promoting impaired autophagy and relieving cartilage degeneration by attenuating inflammatory responses	Restraining of STAT3 and NF-κB signal pathways	[147]
8	Tetrahydrohyperforin	Terpenoids	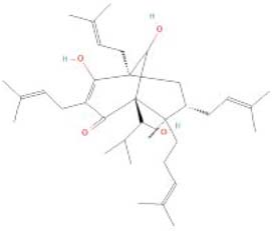	Collagenase solution-induced OA rats	Restoring autophagy and ameliorating OA by alleviating the degeneration of articular cartilage ​	——	[148]
9	Rhoifolin	Flavonoids	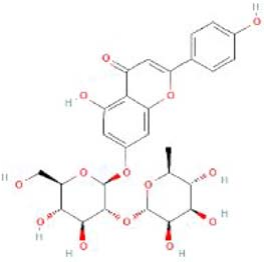	ACLT-induced PTOA rats	Activating autophagy and ameliorating OA by attenuating inflammatory response	Blocking the activation of P38/JNK and PI3K/AKT/mTOR pathways	[144]
10	Quercetin	Flavonoids	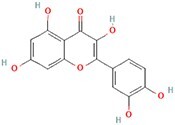	DMM-induced PTOA rats	Activating autophagy and ameliorating OA by suppressing MMP-13 expression and increasing the expression of collagen II and aggrecan	Suppressing RHEB, p-mTOR, p-ULK1 and P62 expression but promoting TSC2 and LC3BII expression	[143]
11	Shikimic acid	Ketones, aldehydes, acids	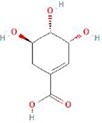	ACLT-induced PTOA rats	Activating autophagy and ameliorating OA by ameliorating cartilage degeneration	Inhibiting MAPK and NF-κB signaling pathways and up-regulating the expression of ATG7, Beclin-1 and LC3	[150]
12	Trehalose	Saccharides	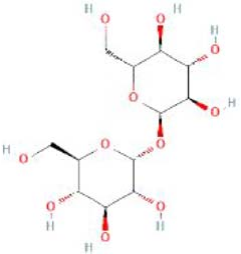	DMM-induced PTOA mice	Promoting autophagic flux and inducing selective autophagy. Ameliorating OA by reducing chondrocyte apoptosis	Ameliorating oxidative stress-mediated mitochondrial damage, ATP level decrease, dynamin-related protein 1 translocation, attenuating mitochondria and endoplasmic reticulum stress-related apoptosis pathway	[151]
13	β-Ecdysterone	Steroids	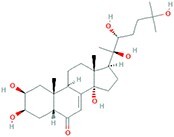	MIA-induced OA rats	Activating autophagy and ameliorating OA by reducing chondrocyte inflammation	Inhibiting the PI3K/AKT/mTOR signaling pathway	[152]

*TRPV5* transient receptor potential cation channel subfamily V member 5, *DMM* destabilization of the medial menisc, *ACLT* anterior cruciate ligament transaction, *mTOR* mammalian target of rapamycin, *AMPK* adenosine 5′-monophosphate (AMP)-activated protein kinase, *MCLT* transection of medial collateral ligament, *PI3K* phosphoinositide 3-kinase, *STAT3* signal transducer and activator of transcription 3, *NF-κB* nuclear factor kappa-B, *JNK* c-Jun N-terminal kinase, *MMP-13* matrix metalloproteinase 13, *RHEB* Ras homolog enriched in brain, *p-ULK1* phospho-unc-51 like autophagy activating kinase 1, *TSC2* tuberous sclerosis complex 2, *LC3* light chain 3, *MAPK* mitogen-activated protein kinase, *ATG7* autophagy related protein 7, *ATP* adenosine triphosphate, *MIA* monosodium iodoacetate. (Structured images from https://pubchem.ncbi.nlm.nih.gov/)

#### Phenols that target autophagy in PTOA

As a polyphenolic drug, punicalagin possesses various pharmacological functions, one of which is autophagy activation and restoration. A previous study revealed that punicalagin can activate autophagy and restore autophagic flux in TBHP-treated chondrocytes, while it ameliorated the degeneration of articular cartilage in mice following DMM [140]. Gallocatechin 3-gallate, an active component of green tea, could activate autophagy and regulate chondrocyte apoptosis by reducing the expression level of mTOR and enhance the expression of microtubule-associated protein light chain 3, BECN1 and p62. In an ACLT-induced OA model, gallocatechin 3-gallate treatment reduced cartilage degradation [141]. Mangiferin is a natural polyphenol. As a potential therapeutic drug for OA, mangiferin could enhance autophagy by activating the AMPK signaling pathway. In a TBHP-induced chondrocyte injury model and mouse PTOA model, Li *et al*. treated mice with mangiferin and reduced chondrocyte apoptosis and cell-matrix degradation [142].

#### Flavonoids that target autophagy in OA

Quercetin is widely present in plants and is able to promote autophagy, mediated by the TSC2-RHBE-mTOR signaling pathway. Lv *et al*. reported that quercetin reduced chondrocyte apoptosis and relieved knee joint injury in a DMM-induced PTOA model [143]. Rhoifolin, a flavonoid extracted from the beech tree, demonstrated autophagy regulation functions through the P38/JNK and PI3K/AKT/mTOR signaling pathways. Yan *et al*. found that rhoifolin could reduce cartilage degradation and delay cartilage damage in a mouse PTOA model [144]. As the main component of epimediums, icariin has various pharmacological effects, including inhibiting the PI3K/AKT/mTOR signaling pathway, up-regulating autophagy genes and reducing chondrocyte apoptosis. In a rat model of OA, icariin treatment significantly alleviated the pathological damage of cartilage [145]. Glabridin is a natural antioxidant small molecule with a strong scavenging effect on free radicals, possessing an autophagy activation function. Glabridin can up-regulate the expression of ECM-related genes including Collagen II, aggrecan, SRY-box 9 and proteoglycan 4, accompanied by an increase of autophagy level *in vitro*. Moreover, intra-articular injection of glabridin protected chondrocytes against apoptosis and alleviated OA progression in ACLT rats, which was partially related to mTOR-mediated autophagy [146].

#### Terpenoids that target autophagy in PTOA

Terpenoids are the most diverse group of secondary metabolites in quantity and structure that are derived from natural sources and have various bioactivities. However, how terpenoids function in OA is still not clear. Alantolactone (ALT), a sesquiterpene lactone compound of terpenoids, is purified from *Inula helenium L*. Previous studies revealed that the ALT can inhibit the phosphorylation of Signal transducer and activator of transcription 3 (STAT3) selectively and restrain its transportation from cytoplasm to the nucleus. STAT3 is activated consistently in IL-1β-induced chondrocytes, while ALT treatment attenuated the inflammatory response and cartilage degeneration by abolishing STAT3 sensitization [147]. Tetrahydrohyperforin, a tetrahydro derivative of hyperforin and a member of the terpenoid group, is one of the main active components of *Hypericum perforatum L*, demonstrated to be able to activate ATG5-depended autophagy. Zhang *et al*. discovered that tetrahydrohyperforin could improve the pathological severity of a joint injury in a rat model that was chemically induced by intra-articular injection of collagenase solution [148]. The role of autophagy regulation in chondrocytes by tetrahydrohyperforin should be further investigated in PTOA models.

#### Other natural extracts that target autophagy in PTOA

Oxoglaucine is the extractor from Magnoliaceae and possesses an autophagy activation function, manifested as accelerating autophagic influx and vesicle formation. In chondrocytes from OA patients and animal OA models, oxoglaucine was found to activate autophagy, delay cartilage matrix degradation and prevent cartilage damage through the TRPV5/calmodulin/CAMK-II pathway [149]. As a natural extract, shikimic acid (SA) shows strong anti-inflammatory properties and can promote autophagy by up-regulating the expression of ATG7, BECN1 and LC3, which was related to the inhibition of MAPK and NF-κB pathways. Moreover, the progression of OA was significantly alleviated by SA in a trauma-induced PTOA rat model [150]. Trehalose was first extracted from Ergot bacteria and is a novel mTOR-independent autophagy inducer. Tang *et al*. found that trehalose ameliorated oxidative stress-mediated mitochondrial damage and ER during the progression of OA after trehalose treatment in a TBHP-induced chondrocyte injury model and a DMM-induced mouse PTOA model, and eventually relieved joint symptoms [151]. β-Ecdysterone, an active ingredient isolated from Achyranthes with broad pharmacological effects, can activate chondrocyte autophagy through regulating the PI3K/AKT/mTOR signaling pathway. In an MIA-induced rat model, the inflammation of chondrocytes was significantly reduced after β-ecdysterone treatment [152]. In future studies, the therapeutic effect of β-ecdysterone on PTOA needs to be evaluated in surgically induced animal models.

From the current research, the natural products regulating autophagy of chondrocytes mostly belong to phenols and flavonoids. The key autophagy genes and signaling pathways regulated by these natural compounds are not exactly the same, suggesting the complexity of their mechanisms of action on chondrocytes. More research needs to be done to explore the pharmacodynamic structures of the active components of these compounds so as to help optimize the structure of existing natural products and make them more specifically regulate autophagy levels in chondrocytes. So far, many natural products have been used in the clinical research of PTOA treatment. With the in-depth research in future, the natural products that target autophagy may be greatly improved in anti-PTOA therapy.

### Physical therapy that targets autophagy in PTOA

Mechanical loading can affect the pathological progression of early and late PTOA. In a mouse OA model, Zheng *et al*. found that mechanical loading promoted the phosphorylation of eukaryotic translation initiation factor 2a to regulate the expression of LC3II/I and p62, and ultimately increased the resistance of chondrocytes to injury [153]. Reasonable exercise can enhance the stability of the knee joint to prevent or delay the occurrence and progression of OA. Li *et al*. found that moderate-intensity exercise promoted autophagy activation through modulating the AMPK/mTOR signaling pathway, resulting in the inhibition of chondrocyte apoptosis and the alleviation of cartilage degeneration *in vitro* [154]. Ozone can inhibit the inflammation of OA and regulate cartilage metabolic balance. In a PTOA model, Xu *et al.* found that ozone could up-regulate the expression level of the autophagy-related protein LC3II, then reduce the inflammation and inhibit MMP-13, which finally reduced cartilage degeneration and pain symptoms [155]. Zhang *et al*. found that 30 min of treadmill exercise promoted autophagy in articular cartilage, decreased inflammation and increased the expression of type II collagen, resulting in the protection of chondrocyte degeneration in a rat model of OA induced by MIA [156]. In general, the activation of autophagy may be an important mechanism of some physical therapies for PTOA patients. We need to further optimize the parameters of physical therapy on the basis of its action mechanism so as to produce better effects in PTOA treatment.

### Biological therapy that targets autophagy in OA

Exosomes are nanoscale extracellular vesicles secreted by cells that can carry biologically active substances such as lipids, proteins and complex RNAs. In a DMM-induced mouse PTOA model, chondrocyte exosomal vesicles inhibited macrophage ATG4B expression via miR449a-5p, then activated inflammation and promoted IL-1β maturation by inhibiting endotoxin-induced autophagy, leading to an increase in synovial inflammation, indicating that targeting exosomal vesicles from inflammatory chondrocytes is a potential strategy for PTOA treatment [157]. In an IL-1β-induced chondrocytes injury model, Wen *et al*. found that human mesenchymal stem cell-derived exosomes (MSC-Exo) upregulated KLF3-AS1 expression, then activated the PI3K/Akt/mTOR signaling pathway and finally inhibited autophagy, mediated by lncRNA (KLF3-AS1). Then, MSC-Exo were found to reduce chondrocyte apoptosis and improve OA symptoms, which is expected to be a potential target for OA therapy [158]. As a novel scavenger of ROS and reactive nitrogen species, dopamine melanin (DM) nanoparticles possess low cytotoxicity and a powerful ability to sequester ROS and reactive nitrogen species. In an animal model of OA, Zhong *et al*. treated mice with intra-articular injection of DM and found that it could alleviate cartilage degradation and reduce inflammatory cytokine release and proteoglycan loss [159]. IL-1β plays a critical role in the progression of OA and is a vital factor for cartilage damage. In a IL-1β-induced chondrocyte injury model and surgically induced mouse PTOA model, Wang *et al*. found that blocking IL-1Ra can facilitate autophagy recovery and delay ECM degradation [160]. MSCs are a kind of early undifferentiated cell with self-renewal, self-replication, unlimited proliferation and multi-directional differentiation potential. MSCs can secrete cytokines, reduce inflammation, reduce tissue cell apoptosis and promote endogenous stem progenitor cell proliferation in sexual tissues and organs. In ACLT and DMM surgically induced rat PTOA models, Chen *et al*. performed phosphate-buffered saline or MSCs-conditioned medium treatment and uncovered that MSC-conditioned medium could enhance autophagy, inhibit chondrocyte apoptosis, protect subchondral bone microstructure and balance the ratio of MMP-13 to TIMP-1 in cartilage, so as to relieve PTOA symptoms [161]. In addition, Zhou *et al*. implanted adipose-derived MSCs into the right knee joint of a surgically induced rat PTOA model and found that they reduced the secretion of pro-inflammatory factors and decreased apoptosis by activating autophagy [162]. Biological therapy, especially related to stem cells and their derivatives, has attracted much attention in PTOA clinical therapy. Activating the autophagy level of chondrocytes by biological products to prevent cartilage degeneration is a potentially feasible strategy for OA treatment, which deserves further exploration in the future.

### Perspective

PTOA is a whole joint disease, involving cartilage, subchondral bone, synovium and other joint tissues. Existing studies have mainly focused on the roles of autophagy in the maintenance of cartilage homeostasis. In recent years, some studies also suggest that autophagy can affect the progression of PTOA by affecting synovial inflammatory cells and subchondral osteoblasts, indicating that autophagy participates in the progression of PTOA by targeting different joint tissues [71,163]. Therefore, more studies are needed to reveal the roles and detailed mechanisms of autophagy during PTOA, so as to more effectively improve the effect of anti-PTOA treatment based on autophagy. With the development of the life sciences and technology, it is believed that we will have a more comprehensive and in-depth understanding of the roles and mechanisms of autophagy in the process of PTOA.

As an important mechanism to maintain cell homeostasis, autophagy in PTOA has attracted increasing attention. Previous studies have mainly focused on the role of macroautophagy in the maintenance of cartilage homeostasis, but other types of autophagy need more attention. Recent studies have shown that mitophagy, a novel manifestation of autophagy, is similarly closely associated with PTOA progression [164]. Mitophagy is generally thought to be a dominant mechanism of mitochondrial quality control. The intact mitochondrial structure is a prerequisite for the survival of chondrocytes [165]. Mitochondrial dysfunction causes metabolic disorders and inflammatory responses in chondrocytes, which in turn contributes to PTOA progression. Zhang *et al*. showed that mitophagy and mitochondrial dynamics are involved in chondrocyte biological stress regulation [166]. Moreover, in an AGE-induced chondrocyte injury model, Tang *et al*. found that bone marrow MSC-Exos inhibited chondrocyte apoptosis and cartilage matrix degradation by drp1-mediated mitophagy [167]. Apart from mitophagy, the roles and mechanisms of other types of autophagy in PTOA should also be given more attention in the future.

Although many strategies have been developed for autophagy evaluation, most of the existing methods are static evaluations and can hardly achieve accurate dynamic monitoring [168]. mRFP-GFP-LC3 is an optional method for the dynamic detection of autophagy, but there is still the problem of inaccurate fluorescent quantification, which needs further improvement and optimization. In addition, the current drugs that activate or inhibit autophagy lack specificity, which may increase the risk of side effects during treatment [169,170]. Therefore, we need more in-depth research on mechanisms of autophagy regulation in order to develop more specific autophagy targeting agents and ultimately provide more effective methods for PTOA therapy in clinic.

## Conclusions

In conclusion, the decrease of autophagy is very common during the PTOA process, and its change is closely related to the occurrence and development of PTOA. As an important mechanism for the maintenance of chondrocyte homeostasis, autophagy can effectively prevent cartilage degeneration by regulating cell death, anabolism and catabolism, hypertrophy and inflammation. The regulatory mechanisms of autophagy during PTOA are highly associated with gene transcription, mRNA stability, protein modification, etc. Some potential therapeutic strategies display their anti-PTOA effects partially through enhancing autophagy. Rational enhancement of autophagy will be a promising mode to prevent the occurrence and development of PTOA in the future. Therefore, we need to further study the mechanisms of autophagy and identify specific targets in PTOA so as to provide effective treatments for clinical PTOA patients.

## Abbreviations

A2AR: Adenosine A2A receptor; ACLT: anterior cruciate ligament transection; ADAMTS: A disintegrin and metalloprotease with thrombospondin motifs; AKT: Serine/threonine kinase 1; AMP: Adenosine monophosphate; AMPK: Adenosine 5-monophosphate-activated protein kinase; APPL1: Adaptor protein, phosphotyrosine interacting with PH domain and leucine zipper 1; ATG: Autophagy-related gene; ATP: Adenosine triphosphate; BECN1: Beclin-1; BFGF: Basic fibroblast growth factor; CCL5: C-C motif chemokine ligand 5; CIR: Cartilage injury related; DDR1: Epithelial discoidin domain-containing; FIP200: FAK family kinase-interacting protein of 200 kDa; HDAC6: Histone deacetylase 6; IGF-1:  Insulin-like growth factor-1; IL-1: Interleukin 1; IL-1Ra: Interleukin 1 receptor alpha; KOA: Knee osteoarthritis; LAMP1: Lysosomal associated membrane protein 1; LC3: Light chain 3; LKB1: Liver kinase B1 homolog; LOXL3: Lysyl oxidase like 3; METTL3: Methyltransferase 3; mLST8: Mammalian lethal with SEC13 protein 8; MMP: Matrix metallopeptidase; MTORC1: Mammalian target of rapamycin complex 1; OA: Osteoarthritis; PE: Phosphatidylethanolamine; PHD: Plant homeodomain; PI3K: Phosphatidylinositol 3-kinase; PIK3R4: Phosphoinositide 3-kinase regulatory subunit 4; PIK3C3: Phosphatidylinositol 3-kinase catalytic subunit type 3; PTOA: Post-traumatic osteoarthritis; PRAS40:40 kDa proline-rich AKT substrate; REDD1: Protein regulated in development and DNA damage response 1; Si-lnc: Small interfering long no-coding; siRNA: Small interfering RNA; SIRT3: Sirtuin 3; SOD2: Superoxide dismutase 2; SQSTM1: Sequestosome-1; STAT3: Signal transducer and activator of transcription 3; TRAF3: TNF receptor associated factor 3; UHRF1: Ubiquitin like with PHD and ring finger domains 1; ULK1: Unc-51-like autophagy activating kinase 1; VEGF: Vascular endothelial growth factor; WIPI2b: WD repeat domain phosphoinositide-interacting protein 2 beta.
